# Vascular plants occurrences in Dokdo Islands, Korea, based on herbarium collections and legacy botanical literature

**DOI:** 10.3897/BDJ.9.e77695

**Published:** 2021-12-20

**Authors:** Chin-Sung Chang, Shin Young Kwon, Hyun Tak Shin, Su-Young Jung, Hui Kim

**Affiliations:** 1 Seoul National University, Seoul, Republic of Korea Seoul National University Seoul Republic of Korea; 2 Korea National Arboretum, Pocheon, Republic of Korea Korea National Arboretum Pocheon Republic of Korea; 3 Mokpo National University, Muan, Republic of Korea Mokpo National University Muan Republic of Korea

**Keywords:** biodiversity, Bromuscatharticus, Dokdo, flora, invasive species, islands, vascular plants

## Abstract

**Background:**

The vascular flora of the Dokdo Islands has been reported, based on primary collections made in 2012 and 2013 and legacy botanical literature. The Dokdo Islands are the remotest islands of Korea, located in the East Sea approximately 87 km from Ulleungdo Islands. They comprise two main volcanic islands, Dongdo (east islands) and Seodo (west islands) and minor islets surrounding the two main islands. This research was conducted to document vascular plant species inhabiting Korea's most inaccessible islands. We present a georeferenced dataset of vascular plant species collected during field studies on the Dokdo Islands over the past seven decades.

**New information:**

In the present inventory of the flora of Dokdo, there are listed 108 species belonging to 78 genera and 39 families, including 93 native species and 15 newly human-induced naturalised species for these Islands' flora. The Poaceae and Asteraceae families are the most diverse, with 22 and 15 taxa, respectively. Some of the previously-listed taxa were not found on Dokdo probably because they are rare and the limited time did not allow collectors to find rare species. The spread of introduced species, especially the invasive grass *Bromuscatharticus* Vahl., affects several native species of Dokdo flora.

## Introduction

Biodiversity researchers have identified critical gaps in spatial, temporal and taxonomic coverage of biodiversity observations highlighting barriers to effective data collection, open access and analysis (Amano et al. 2016, Wetzel et al. 2018). To bridge these gaps, biodiversity data must suit the demands of multiple groups, including scientists, policy-makers and data contributors (Taylor et al. 2017). Several biodiversity data researchers have emphasised taking the lead in developing new measures. Options like open access publishing with conventional licences accessibility through major biodiversity platforms, such as GBIF, can be used (Faith et al. 2013). The next solution is offering data providers incentives, such as the option to publish in peer-reviewed data journals (Chavan and Penev 2011). Biodiversity data providers should become better data stewards, with a comprehensive understanding of metadata, best data management practices and plans for data archiving and preservation (Hartter et al. 2013, Penev et al. 2017). However, data stewardship takes time and resources and data providers cannot be data stewards without sufficient resources and support. The evolution of data stewardship culture causes biodiversity informatics challenges to emerge as data volume and precision increase. Biodiversity data scientists propose that data providers and stakeholders confront current challenges prividing them with detailed recommendations (Ariño et al. 2016).

Geographical location and security level are the main factors causing spatial gaps (Ariño et al. 2016). As biodiversity information is closely related to the temporal and spatial variation in surveying effort, Wallacean shortfall is specifically critical in remote and inaccessible areas (Hortal et al. 2008, Boakes et al. 2010). Sampling certain places better than others is inevitable given the accessibility differences between localities (Rodrigues et al. 2010); therefore, distribution data tend to be heavily biased with historical collection patterns, collation and biodiversity data accumulation (Rodrigues et al. 2010, Meyer et al. 2015). To effectively bridge spatial gaps, it is essential to comprehend the causes for data shortage in some regions. In the case of Banco de Datos de Biodiversidad de Canaris (BIOTA-Canarias, Hortal et al. 2007), it stated that the lack of completeness or large gaps in their spatial coverage compromises their future utility. The previously collected data have limited utility because the data lack detail and geographical coverage is not exhaustive (Soberón et al. 2007). Biodiversity data scientists encourage exhaustive compilation of all available information with sufficient quality and detail (Hortal et al. 2008).

The Dokdo Islands are the most inaccessible islands in Korea, located at 37°14'26.8"N and 131°52'10.4"E, belonging to an administrative district that includes the Ulleung Islands. Since the first botanical survey (Lee 1952), seventy years of sporadic observations have waited to be mobilised to accessible biodiversity data (Jung et al. 2014). This study produces an exhaustive and reliable list of vascular plants from the Dokdo Islands, based on reference herbarium specimens collected in the field and the occurrence data available in the papers (Kim and Lee 2021).

## General description

### Purpose

This research focused on the digitisation of plant distribution data on Dokdo Islands acquired by botanists on occasional expeditions to the Islands between 1947 and 2018. These data offer a promising tool to help guide the biodiversity management and conservation of these highly inaccessible island ecosystems.

## Project description

### Title

Vascular plants occurrences in Dokdo Islands, Korea, based on herbarium collections and legacy botanical literature.

### Personnel

The datasets were digitised by Hui Kim (data manager), Su-Young Jung was the resource creator and Shin Young Kwon, Hyun Tak Shin and Chin-Sung Chang were the content providers. Chin-Sung Chang checked taxonomic changes and georeferencing. S.Y. Jung conducted the field works for two years, from April 2012 to September 2013, collaborating with members from Korea National Arboretum (Jung et al. 2014). S.Y. Jung did preliminary *in situ* identifications. S.Y. Jung, Hui Kim and Chin-Sung Chang conducted the final species identification.

### Study area description

The small islands of Dokdo are volcanic rocks formed in the Cenozoic era, more specifically 4.6-2.5 million years ago, having a formation mechanism similar to underwater islands (Jo et al. 2021, Kim et al. 2013). The Dokdo Volcano rises roughly 2,100 m a.s.l. and has a diameter of more than 10 km (Song et al. 2017). The Islands have a butterfly wing shape, a relatively steep terrain, a peak elevation of 168 m a.s.l. and a surface area of 18.7 hectares (Fig. 1). The Dokdo Islands consist of two main islets, Seodo and Dongdo, with numerous surrounding rocks. Sedo has multiple berth and tracking routes access points and flora surveys and collections are possible over a comparatively large area. Since Dongdo is more difficult to access by boat, it is challenging to investigate the surface, except there are fewer primary species occurrence data in a few points. Dokdo Islands had a mean annual temperature of 13.8°C, mean annual precipitation of 589 mm, an absolute minimum temperature of -6.4°C and an absolute maximum temperature of 28.2°C. According to meteorologists, automatic weather systems underestimate the amount of snowfall, thereby resulting in missing data (Kim and Park 2017).

## Sampling methods

### Study extent

The Dokdo Islands are the most inaccessible islands in Korea, located at 37°14'26"N and 131°52'05"E, belonging to an administrative district that includes the Ulleung Islands.

### Sampling description

The vascular plant occurrence data, treated in this study, were compiled using fieldwork from 2012 to 2013 and botanical legacy articles from 1947 to 2018. Herbarium surveys were conducted in two Herbaria, including SNUA (Seoul National University, College of Agriculture, herbarium acronym following Index Herbariorum) and KH (Korea National Arboretum). In addition to the authors’ collections, datasets on vascular plant occurrences in Dokdo Islands were digitised from several manuscripts in a heterogeneous format (Lee 1952, Lee and Joo 1958, Lee 1978, Sun et al. 2002, Hyun and Kwon 2006, Lee et al. 2007, Park and Lee 2008, Park et al. 2010, Song and Park 2012, Jung et al. 2014, Park et al. 2014, Kim and Lee 2016, Park et al. 2016, Park et al. 2017, Park et al. 2018, Table 1). References to the published literature, from which data were obtained for the occurrence data compilation, are presented in the bibliography section of the metadata.

### Quality control

The Dokdo Islands occurrence dataset was manually digitised from scanned documents of the original papers. The quality control processes of biodiversity data management were based on the principles of data quality by Chapman (2005) . Scientific names and locality names in the digitised datasets were retained exactly as in the original papers. The authors used the provisional checklist of vascular plants for the Korea Peninsula Flora to determine the accepted names (Chang et al. 2014). All scientific names were cross-checked and taxonomically updated using the taxonomic module of Botanical Research and Herbarium Management System (BRAHMS; Pouwer et al. 2008); more details on the digitisation steps, structure of the data and quality control measures are presented below.

### Step description

1. The content providers carefully reviewed individual floristic publications to manage the irregularity in the format of historical papers. All occurrence records were merged into a spreadsheet, which contained the original species names recorded at the location. In this digitisation stage, obvious typographic errors were corrected. Accepted taxon names and taxonomic classification derived from the local checklist (Chang et al. 2014) were included in the spreadsheet. The result of the above digitisation steps was 838 records with 25 columns containing occurrence data of 108 vascular plant taxa.

2. MS Access was used to create the BRAHMS database layout. All specimen and occurrence information were recorded in the BRAHMS database of the T.B. Lee Herbarium.

3. In the literature data, we frequently encountered several uncertain dates of field works, for instance, 13 July 2017; 26 September 2017; 17 April 2018; 19-20 June 2018; 18 September 2018, for 68 collections by Park et al. (2018). When the collection date was written as “several dates,” we transcribed the last dates of field works (day, month and year) and provided the full interval date in the eventDate field and the rest of the general information in the verbatimEventDate field. Park and Lee (2008) and Park et al. (2017) published the floristic list of Dokdo Islands with many vascular plant pictures. As these authors did not provide the collection information, the publication year was used as the year of events.

4. All occurrence records without coordination were georeferenced, either from the coordinates provided in the paper or from the geographic description of the localities. The coordinate uncertainty in metres for each occurrence was estimated employing the algorithm of Wieczorek et al. (2010).

5. Occurrence data in BRAHMS could be easily exported in various formats, including Darwin Core for uploading to the EABCN IPT. The Darwin Core standard was applied to the BRAHMS extract/query file structure to accommodate the relevant information extracted from the publications.

## Geographic coverage

### Description

Dokdo Islands, Ulleung-gun, Geongsangbuk-do, the Republic of Korea (approximately 37°14'26"N, 131°52'5"E)

### Coordinates

37.225 and 37.255 Latitude; 131.823 and 131.9 Longitude.

## Taxonomic coverage

### Description

All vascular plants were identified to infraspecific level. This dataset contains distribution information for 108 vascular plant species belonging to 39 families (Table 2).

## Traits coverage

### Data coverage of traits

PLEASE FILL IN TRAIT INFORMATION HERE

## Temporal coverage

### Notes

Sampling was conducted on several occasions in the period between 1947 and 2018.

## Usage licence

### Usage licence

Creative Commons Public Domain Waiver (CC-Zero)

### IP rights notes

This work is licensed under a Creative Commons Attribution (CC-BY) 4.0 License.

## Data resources

### Data package title

Vascular plant occurrences in Dokdo Islands, Korea, based on herbarium collections and legacy botanical literature.

### Resource link

https://www.gbif.org/dataset/37663a11-6c27-4b72-a3bc-75c9dab75a83

### Alternative identifiers

http://61.82.48.86:8080/ipt-2.4.2/resource?r=dokdo_flora

### Number of data sets

1

### Data set 1.

#### Data set name

Vascular plant occurrences in Dokdo Islands, Korea, based on herbarium collections and legacy botanical literature.

#### Data format

Darwin Core Archive

#### Number of columns

37

#### Download URL

https://www.gbif.org/dataset/37663a11-6c27-4b72-a3bc-75c9dab75a83

#### Description

The present project was focused on digitising the data on plant distribution on Dokdo Islands, collected between 1947 and 2018 by botanists taking part in occasional expeditions to the Islands. These data are expected to contribute to the biodiversity management and conservation of these highly inaccessible island ecosystems.

**Data set 1.** 

Column label	Column description
occurrenceID	An identifier for the Occurrence (as opposed to a particular digital record of the occurrence). In the absence of a persistent global unique identifier, construct one from a combination of identifiers in the record that will most closely make the occurrenceID globally unique.
recordedBy	A list (concatenated and separated) of names of people, groups or organisations responsible for recording the original Occurrence. The primary collector or observer, especially the one who applies a personal identifier (recordNumber), should be listed first.
type	The nature or genre of the resource.
basisOfRecord	The specific nature of the data record.
institutionCode	The name (or acronym) in use by the institution having custody of the object(s) or information referred to in the record.
recordNumber	An identifier given to the Occurrence at the time it was recorded. Often serves as a link between field notes and an Occurrence record, such as a specimen collector's number.
day	The integer day of the month on which the Event occurred.
month	The integer month in which the Event occurred.
year	The four-digit year in which the Event occurred, according to the Common Era Calendar.
eventDate	The date-time or interval during which an Event occurred. For occurrences, this is the date-time when the event was recorded. Not suitable for a time in a geological context.
verbatimEventDate	The verbatim original representation of the date and time information for an Event.
country	The name of the country or major administrative unit in which the Location occurs.
countryCode	The standard code for the country in which the Location occurs.
stateProvince	The name of the next smaller administrative region than country (state, province, canton, department, region etc.) in which the Location occurs.
county	The full, unabbreviated name of the next smaller administrative region than stateProvince (county, shire, department etc.) in which the Location occurs.
locality	The specific description of the place. Less specific geographic information can be provided in other geographic terms (higherGeography, continent, country, stateProvince, county, municipality, waterBody, island, islandGroup). This term may contain information modified from the original to correct perceived errors or standardise the description.
decimalLatitude	The geographic latitude (in decimal degrees, using the spatial reference system given in geodeticDatum) of the geographic centre of a Location. Positive values are north of the Equator, negative values are south of it. Legal values lie between -90 and 90, inclusive.
decimalLongitude	The geographic longitude (in decimal degrees, using the spatial reference system given in geodeticDatum) of the geographic centre of a Location. Positive values are east of the Greenwich Meridian, negative values are west of it. Legal values lie between -180 and 180, inclusive.
geodeticDatum	The ellipsoid, geodetic datum or spatial reference system (SRS) upon which the geographic coordinates given in decimalLatitude and decimalLongitude are based.
coordinateUncertaintyInMeters	The horizontal distance (in metres) from the given decimalLatitude and decimalLongitude describing the smallest circle containing the whole of the Location. Leave the value empty if the uncertainty is unknown, cannot be estimated or is not applicable (because there are no coordinates). Zero is not a valid value for this term.
georeferencedBy	A list (concatenated and separated) of names of people, groups or organisations who determined the georeference (spatial representation) for the Location.
identifiedBy	A list (concatenated and separated) of names of people, groups or organisations who assigned the Taxon to the subject.
scientificName	The full scientific name, with authorship and date information, if known. When forming part of an Identification, this should be the name in lowest level taxonomic rank that can be determined. This term should not contain identification qualifications, which should instead be supplied in the IdentificationQualifier term.
kingdom	The full scientific name of the kingdom in which the taxon is classified.
phylum	The full scientific name of the phylum or division in which the taxon is classified.
class	The full scientific name of the class in which the taxon is classified.
order	The full scientific name of the order in which the taxon is classified.
family	The full scientific name of the family in which the taxon is classified.
taxonomicStatus	The status of the use of the scientificName as a label for a taxon. Requires taxonomic opinion to define the scope of a taxon. Rules of priority then are used to define the taxonomic status of the nomenclature contained in that scope, combined with the expert's opinion. It must be linked to a specific taxonomic reference that defines the concept.
acceptedNameUsage	The full name, with authorship and date information, if known, of the currently accepted taxon.
vernacularName	A common or vernacular name.
genus	The full scientific name of the genus in which the taxon is classified.
specificEpithet	The name of the first or species epithet of the scientificName.
scientificNameAuthorship	The authorship information for the scientificName formatted according to the conventions of the applicable nomenclaturalCode.
infraspecificEpithet	The name of the lowest or terminal infraspecific epithet of the scientificName, excluding any rank designation.
taxonRank	The taxonomic rank of the most specific name in the scientificName.
nomenclaturalCode	The nomenclatural code (or codes in the case of an ambiregnal name) under which the scientificName is constructed.

## Additional information

**Figure 3.**
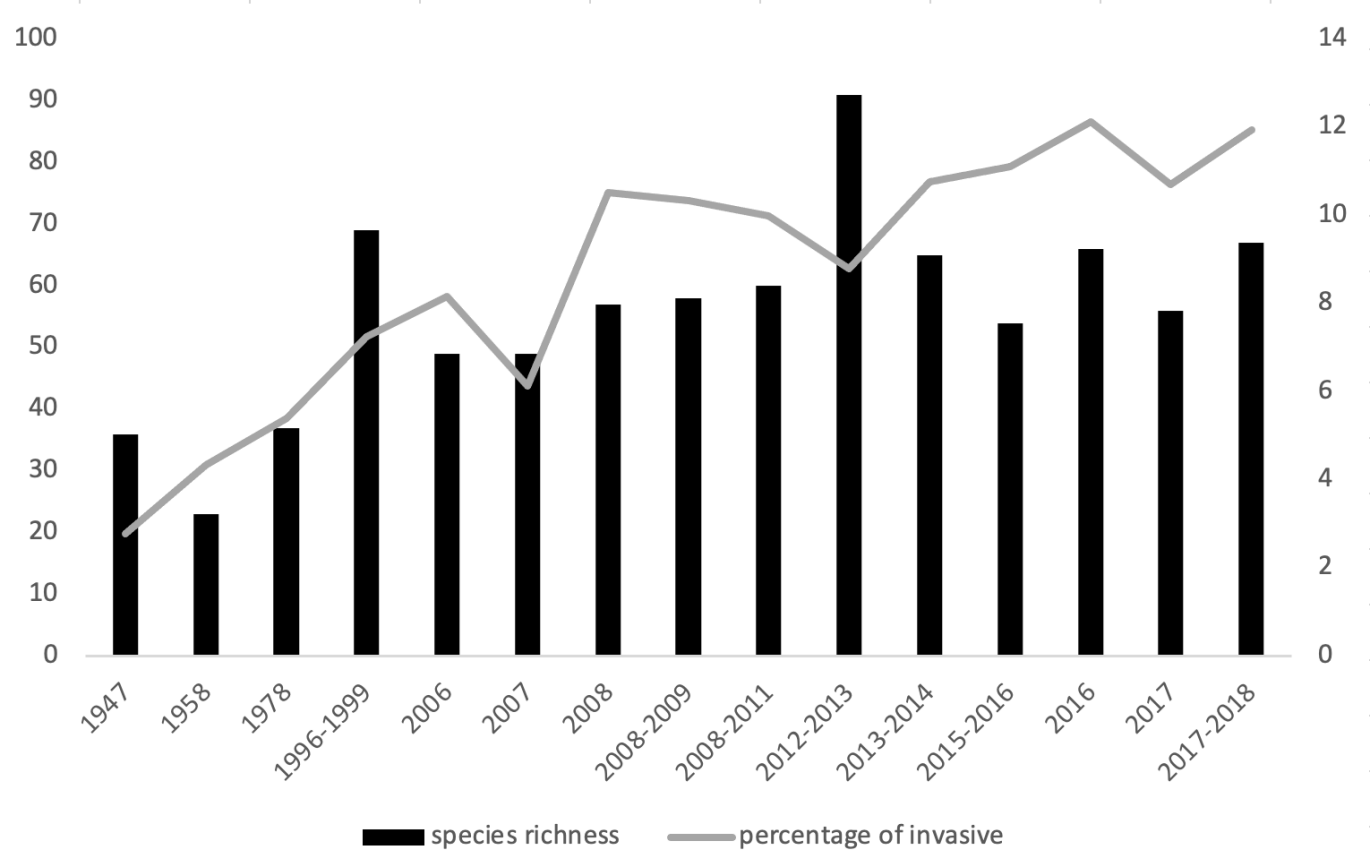
Species richness (histogram, left) and percentage of invasive species (line, right) in Dokdo Islands.

During the seventy years’ observation period (1947-2018), 108 taxa from 39 families were observed. Almost all were flowering plants (only one fern species and one conifer species were recorded), mostly Magnoliopsida (98%). This paper includes 91 specimens and 747 occurrence data of vascular plants recorded in Dokdo Islands regarding 108 taxa identified to infraspecific level. The confirmed species comprise 75 dicots and 31 monocots, one gymnosperm and a non-seed plant (Pteridophytes) species. Most species are native, including *Cyrtomiumfalcatum* (L.f.) C.Presl, *Dianthuslongicalyx* Miq., *Tetragoniatetragonoides* (Pall.) Kuntze, *Fallopiasachalinensis* (F.Schmidt) Ronse Decr., *Lysimachiamauritiana* Lam., *Sedumoryzifolium* Makino, CorydalisheterocarpaSiebold & Zucc.var.japonica (Franch. & Sav.) Ohwi and *Orobanchecoerulescens* Stephan (Fig. 2). The data collected during the last seven decades indicate continuous expansion of invasive species and increase in their richness (Fig. 3). For instance, *Bromuscatharticus* Vahl, *Sonchusasper* (L.) Hill., *Seneciovulgaris* L., *Setariapumila* (Poir.) Roem. & Schult. and *Lycopersiconesculentum* Mill. are the most rapidly expanding aliens in the last decade, threatening native flora (Table 2, Fig. 3). Park et al. (2017) identified increased human visitation as a major predictor of the spatial distribution of invasive species in the flora of Dokdo Islands, assuming a positive relationship between human activities and alien plant species richness. The major threatening species, especially the invasive grass, *Bromuscatharticus* Vahl., affects several native species. Regarding the colonisation status, 14% of total species richness were invasive species and 86% were native to the Korean Peninsula and adjacent islands.

## Figures and Tables

**Figure 1a.**
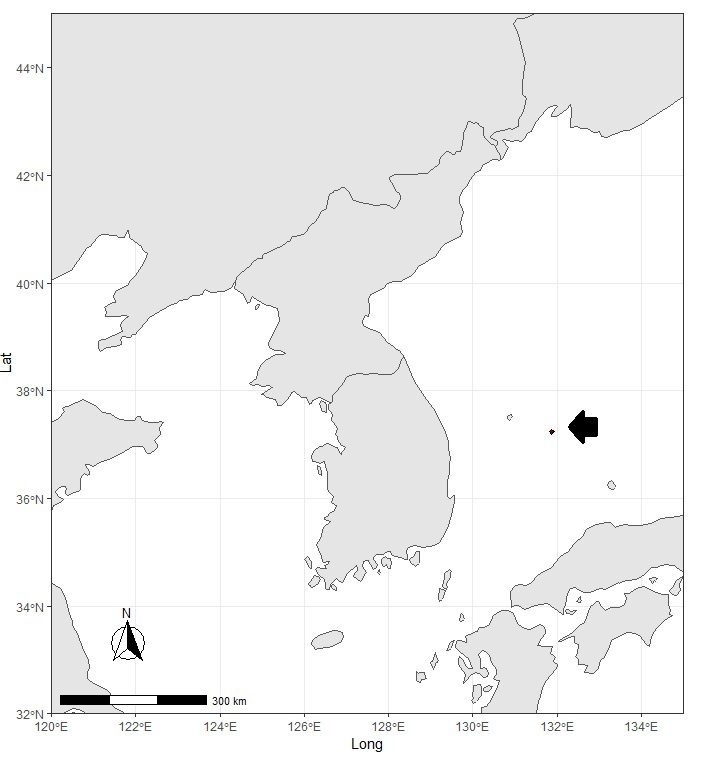
Black arrow points to the location of Dokdo Islands.

**Figure 1b.**
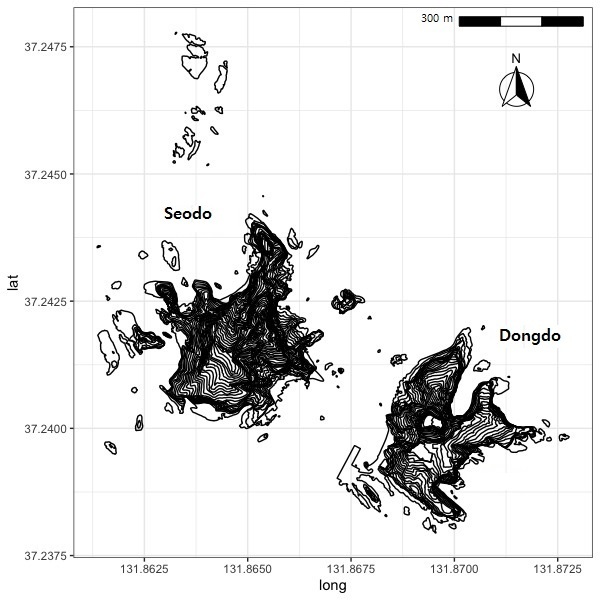
Topography of Dokdo Islands.

**Figure 2a.**
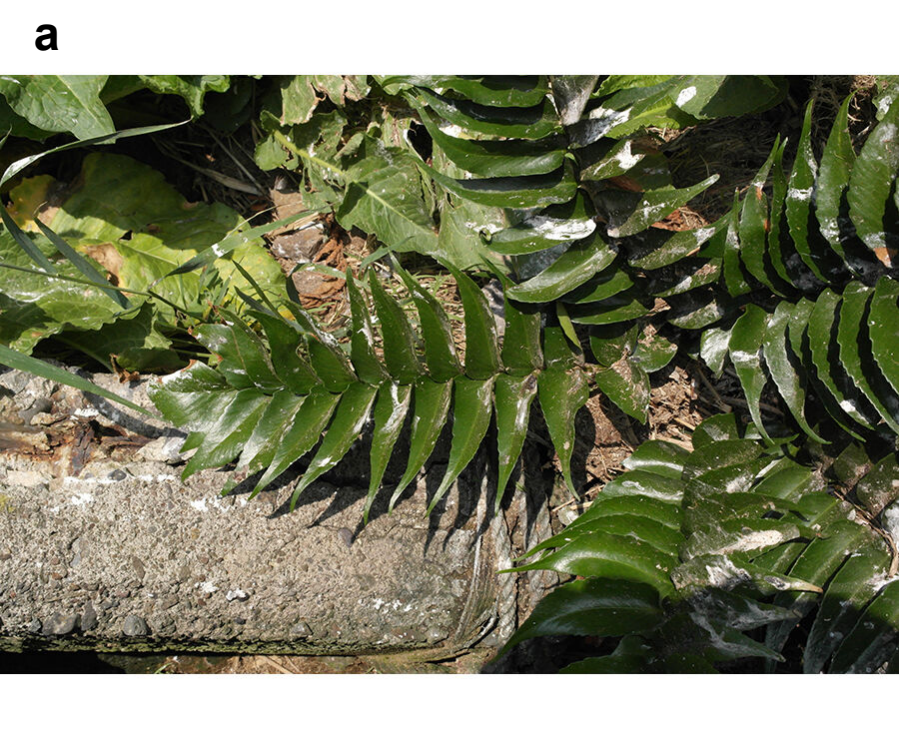
*Cyrtomiumfalcatum* (L.f.) C.Presl

**Figure 2b.**
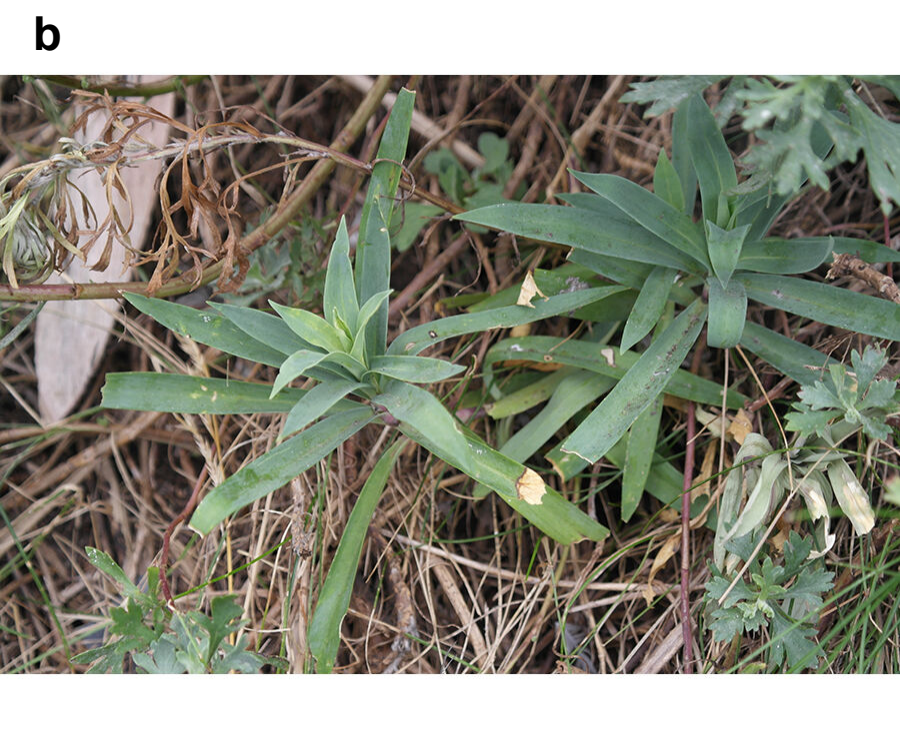
*Dianthuslongicalyx* Miq.

**Figure 2c.**
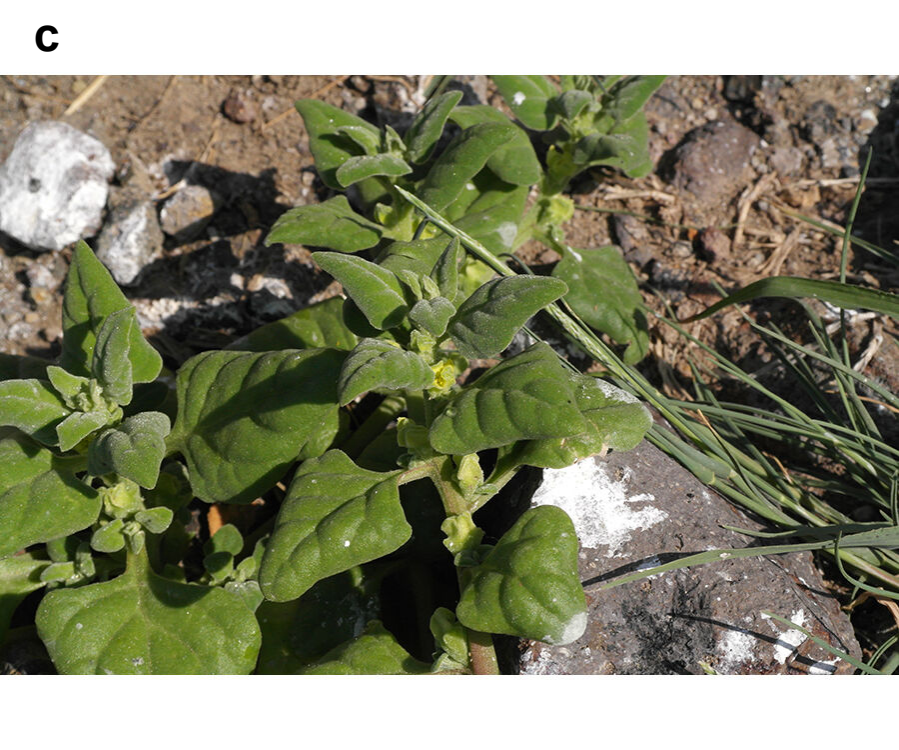
*Tetragoniatetragonoides* (Pall.) Kuntze

**Figure 2d.**
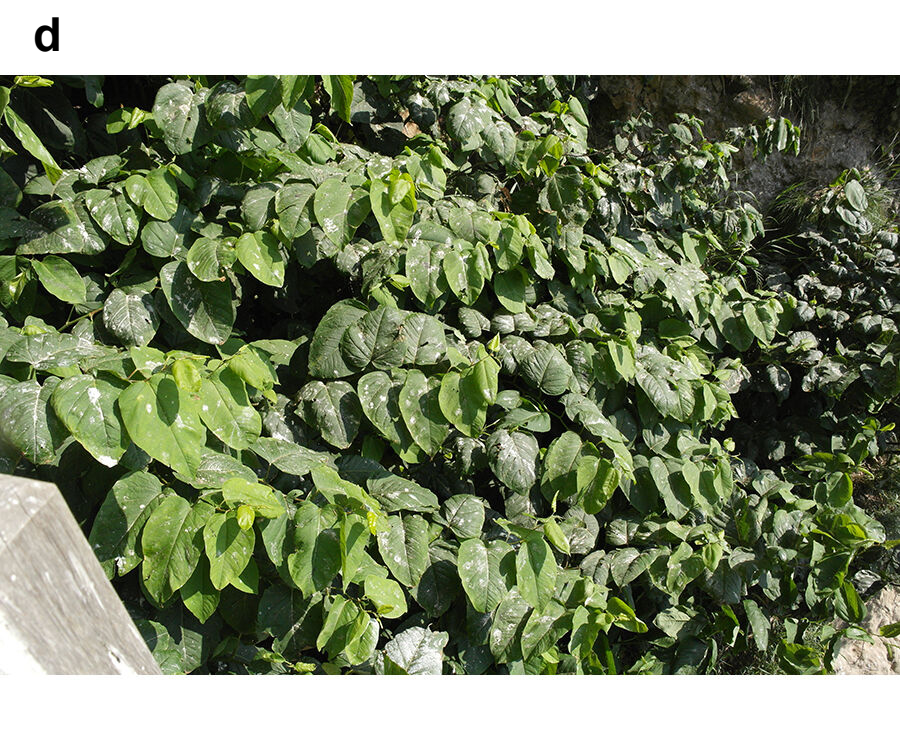
*Fallopiasachalinensis* (F.Schmidt) Ronse Decr.

**Figure 2e.**
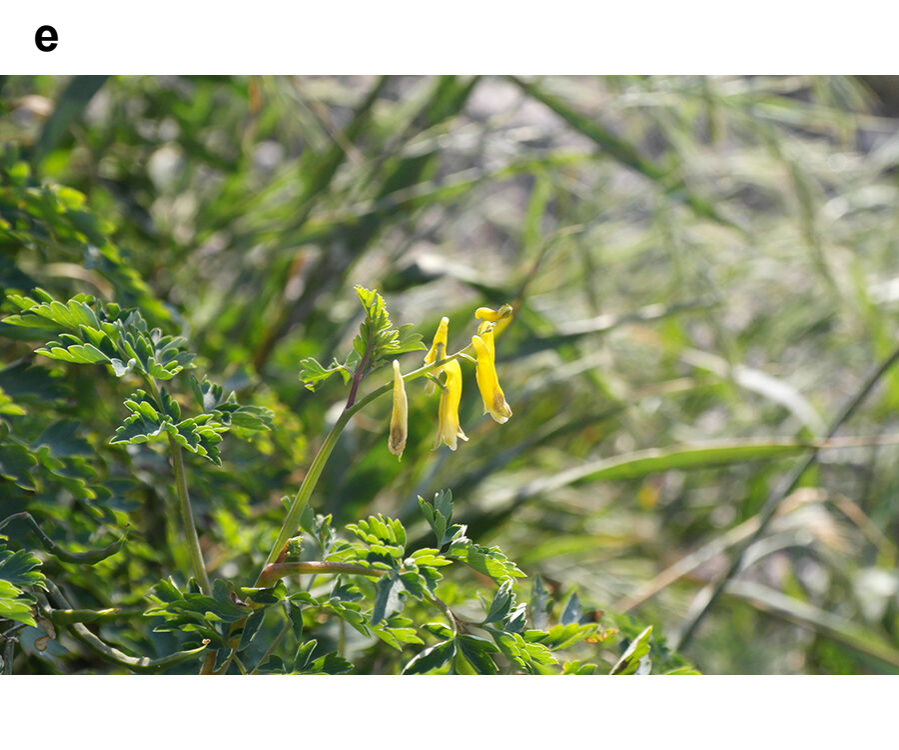
CorydalisheterocarpaSiebold & Zucc.var.japonica (Franch. & Sav.) Ohwi

**Figure 2f.**
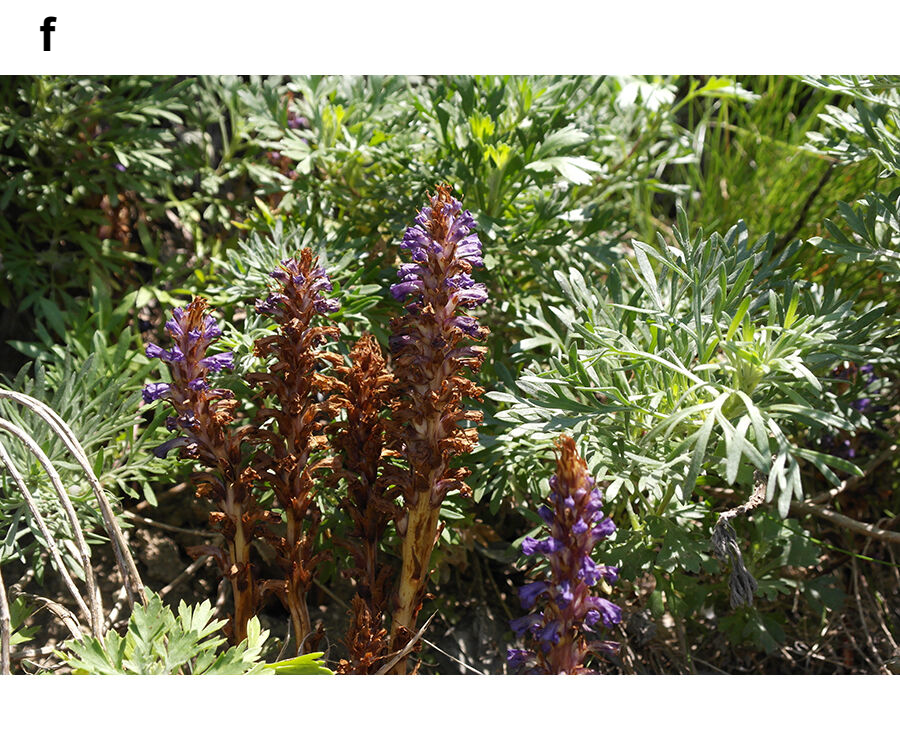
*Orobanchecoerulescens* Stephan.

**Table 1.** Data sources for the dataset of vascular plants occurrences in Dokdo Islands.

Data source	Type of occurrence data	Number of occurrences	Field year
Lee 1952	Literature	36	1947
Lee and Joo 1958	Literature	23	1958
Lee 1978	Herbarium	37	1978
Sun et al. 2002	Literature	69	1996-1999
Hyun and Kwon 2006	Literature	49	2006
Lee et al. 2007	Literature	49	2007
Park and Lee 2008	Literature	57	2008
Park et al. 2010	Literature	58	2008-2009
Song and Park 2012	Literature	60	2008-2011
Jung et al. 2014	Herbarrium/ Literature	91	2012-2013
Park et al. 2014	Literature	65	2013-2014
Kim and Lee 2016	Literature	54	2015-2016
Park et al. 2016	Literature	56	2016
Park et al. 2017	Literature	66	2017
Park et al. 2018	Literature	68	2017-2018
Total		838	

**Table 2.** Classification of species according to the criteria of Family, Habitat and Geographical origin is based on Chang et al. (2014).

**Number**	**SPECIES**	**FAMILY**		**HABIT**	**Geographic Origin**
1	*Tetragoniatetragonoides* (Pall.) Kuntze	Aizoaceae		Herb	Native
2	*Achyranthesbidentata* Blume	Amaranthaceae		Herb	Native
3	AchyranthesbidentataBlumevar.japonica Miq.	Amaranthaceae		Herb	Native
4	*Cnidiumjaponicum* Miq.	Apiaceae		Herb	Native
5	*Oenanthejavanica* (Blume) DC.	Apiaceae		Herb	Native
6	*Metaplexisjaponica* (Thunb.) Makino	Apocynaceae		Herb	Native
7	*Artemisiacodonocephala* Diels	Asteraceae		Herb	Native
8	*Artemisiaindica* Willd.	Asteraceae		Herb	Native
9	*Artemisiajaponica* Thunb.	Asteraceae		Herb	Native
10	*Artemisiakoidzumii* Nakai	Asteraceae		Herb	Native
11	*Artemisiamontana* (Nakai) Pamp.	Asteraceae		Herb	Native
12	*Artemisiascoparia* Waldst. & Kit.	Asteraceae		Herb	Native
13	*Asterspathulifolius* Maxim.	Asteraceae		Herb	Native
14	*Dendranthemanaktongense* (Nakai) Tzvelev	Asteraceae		Herb	Native
15	*Farfugiumjaponicum* (L.) Kitam.	Asteraceae		Herb	Native
16	*Seneciovulgaris* L.	Asteraceae		Herb	Introduced
17	*Sonchusasper* (L.) Hill	Asteraceae		Herb	Native
18	*Sonchusbrachyotus* DC.	Asteraceae		Herb	Native
19	*Sonchusoleraceus* L.	Asteraceae		Herb	Native
20	*Taraxacumplatycarpum* Dahlst.	Asteraceae		Herb	Native
21	*Youngiajaponica* (L.) DC.	Asteraceae		Herb	Native
22	*Arabisserrata* Franch. & Sav.	Brassicaceae		Herb	Native
23	*Arabisstelleri* DC.	Brassicaceae		Herb	Native
24	*Brassicajuncea* (L.) Czern.	Brassicaceae		Herb	Introduced
25	*Capsellabursa-pastoris* (L.) Medik.	Brassicaceae		Herb	Native
26	*Lepidiumvirginicum* L.	Brassicaceae		Herb	Introduced
27	*Raphanussativus* L.	Brassicaceae		Herb	Introduced
28	*Campanulapunctata* Lam.	Campanulaceae		Herb	Native
29	*Loniceramorrowii* A.Gray	Caprifoliaceae		Shrub	Native
30	*Dianthuslongicalyx* Miq.	Caryophyllaceae		Herb	Native
31	*Gypsophilaoldhamiana* Miq.	Caryophyllaceae		Herb	Native
32	*Saginajaponica* (Sw.) Ohwi	Caryophyllaceae		Herb	Native
33	*Saginamaxima* A.Gray	Caryophyllaceae		Herb	Native
34	*Stellariaaquatica* (L.) Scop.	Caryophyllaceae		Herb	Native
35	*Stellariamedia* (L.) Vill.	Caryophyllaceae		Herb	Native
36	*Stellarianeglecta* Weihe	Caryophyllaceae	Herb		Native
37	*Euonymushamiltonianus* Wall.	Celastraceae		Shrub	Native
38	*Euonymusjaponicus* Thunb.	Celastraceae		Shrub	Native
39	*Atriplexgmelinii* C.A.Mey. ex Bong.	Chenopodiaceae		Herb	Native
40	*Atriplexsubcordata* Kitag.	Chenopodiaceae		Herb	Native
41	*Chenopodiumalbum* L.	Chenopodiaceae		Herb	Native
42	*Chenopodiumgiganteum* D.Don	Chenopodiaceae		Herb	Native
43	*Chenopodiumglaucum* L.	Chenopodiaceae		Herb	Introduced
44	*Chenopodiumstenophyllum* (Makino) Koidz.	Chenopodiaceae		Herb	Native
45	*Hypericumerectum* Thunb.	Clusiaceae		Herb	Native
46	*Commelinacommunis* L.	Commelinaceae		Herb	Native
47	*Calystegiasoldanella* (L.) R.Br.	Convolvulaceae		Herb	Native
48	*Ipomoeapurpurea* (L.) Roth	Convolvulaceae		Herb	Introduced
49	*Phedimusmiddendorffianus* (Maxim.) 't Hart	Crassulaceae		Herb	Native
50	*Sedumjaponicum* Siebold ex Miq.	Crassulaceae		Herb	Native
51	*Sedumkamtschaticum* Fisch. & C.A.Mey.	Crassulaceae		Herb	Native
52	*Sedumoryzifolium* Makino	Crassulaceae		Herb	Native
53	*Cucumismelo* L.	Cucurbitaceae		Herb	Introduced
54	*Cyperusmicroiria* Steud.	Cyperaceae		Herb	Native
55	*Cyrtomiumfalcatum* (L.f.) C.Presl	Dryopteridaceae		Herb	Native
56	*Elaeagnusmacrophylla* Thunb.	Elaeagnaceae		Liana	Native
57	*Machilusthunbergii* Siebold & Zucc. ex Meisn.	Lauraceae		Tree	Native
58	*Alliumfistulosum* L.	Liliaceae		Herb	Introduced
59	*Alliummacrostemon* Bunge	Liliaceae		Herb	Native
60	*Asparaguscochinchinensis* (Lour.) Merr.	Liliaceae		Herb	Native
61	*Asparagusschoberioides* Kunth	Liliaceae		Herb	Native
62	*Liliumlancifolium* Thunb.	Liliaceae		Herb	Native
63	*Liriopemuscari* (Decne.) L.H.Bailey	Liliaceae		Herb	Native
64	*Maianthemumdilatatum* (A.W.Wood) A.Nelson & J.F.Macbr.	Liliaceae		Herb	Native
65	*Hibiscussyriacus* L.	Malvaceae		Shrub	Introduced
66	*Cocculusorbiculatus* (L.) DC.	Menispermaceae		Liana	Native
67	*Orobanchecoerulescens* Stephan	Orobanchaceae		Herb	Native
68	*Oxaliscorniculata* L.	Oxalidaceae		Herb	Native
69	*Oxalisstricta* L.	Oxalidaceae		Herb	Native
70	CorydalisheterocarpaSiebold & Zucc.var.japonica (Franch. & Sav.) Ohwi	Papaveraceae		Herb	Native
71	*Pinusthunbergii* Parl.	Pinaceae		Tree	Native
72	*Plantagoasiatica* L.	Plantaginaceae		Herb	Native
73	*Bromuscatharticus* Vahl	Poaceae		Herb	Introduced
74	*Cleistogeneshackelii* (Honda) Honda	Poaceae		Herb	Native
75	*Digitariaciliaris* (Retz.) Koeler	Poaceae		Herb	Native
76	*Digitariaradicosa* (J.Presl) Miq.	Poaceae		Herb	Native
77	*Digitariaviolascens* Link	Poaceae		Herb	Native
78	*Echinochloacrus-galli* (L.) P.Beauv.	Poaceae		Herb	Native
79	*Echinochloaoryzoides* (Ard.) Fritsch	Poaceae		Herb	Native
80	*Eleusineindica* (L.) Gaertn.	Poaceae		Herb	Native
81	*Elymuskamoji* (Ohwi) S.L.Chen	Poaceae		Herb	Native
82	*Festucaovina* L.	Poaceae		Herb	Native
83	*Festucarubra* L.	Poaceae		Herb	Native
84	*Imperatacylindrica* (L.) Raeusch.	Poaceae		Herb	Native
85	*Miscanthussinensis* Andersson	Poaceae		Herb	Native
86	*Pennisetumglaucum* (L.) R.Br.	Poaceae		Herb	Native
87	*Phragmitesjaponicus* Steud.	Poaceae		Herb	Native
88	*Poaannua* L.	Poaceae		Herb	Native
89	*Poapratensis* L.	Poaceae		Herb	Native
90	*Puccinellianipponica* Ohwi	Poaceae		Herb	Native
91	*Setariafaberi* R.A.W.Herrm.	Poaceae		Herb	Native
92	*Setariapumila* (Poir.) Roem. & Schult.	Poaceae		Herb	Introduced
93	*Setariaviridis* (L.) P.Beauv.	Poaceae		Herb	Introduced
94	*Zoysiajaponica* Steud.	Poaceae		Herb	Native
95	*Fallopiasachalinensis* (F.Schmidt) Ronse Decr.	Polygonaceae		Herb	Native
96	*Persicarialongiseta* (Bruijn) Kitag.	Polygonaceae		Herb	Native
97	*Polygonumaviculare* L.	Polygonaceae		Herb	Native
98	*Rumexcrispus* L.	Polygonaceae		Herb	Introduced
99	*Rumexjaponicus* Houtt.	Polygonaceae		Herb	Native
100	*Portulacaoleracea* L.	Portulacaceae		Herb	Native
101	*Lysimachiamauritiana* Lam.	Primulaceae		Herb	Native
102	*Ranunculussilerifolius* H.Lév.	Ranunculaceae		Herb	Native
103	*Rubusphoenicolasius* Maxim.	Rosaceae		Shrub	Native
104	*Lycopersiconesculentum* Mill.	Solanaceae		Herb	Introduced
105	*Solanumamericanum* Mill.	Solanaceae		Herb	Introduced
106	*Camelliajaponica* L.	Theaceae		Shrub	Native
107	*Violakusanoana* Makino	Violaceae		Herb	Native
108	Ampelopsisglandulosa(Wall.)Momiy.var.heterophylla (Thunb.) Momiy.	Vitaceae		Liana	Native
